# Supplementary material for: Variation in Human Milk Composition Is Related to Differences in Milk and Infant Fecal Microbial Communities
Source: Microorganisms. 2021 May 27;9(6):1153. doi: 10.3390/microorganisms9061153 (PMC8230061; doi:10.3390/microorganisms9061153)
Supplement: Supplementary file 1 [file microorganisms-09-01153-s001.zip › 210520 INSPIRE_milk_composition_and_microbes SUPPLEMENT.pdf]

Supplementary Materials

Variation in human milk composition is related to differences in milk and infant fecal microbial communities

Figure S1: Relative abundances of the initial top 25 bacterial genera in milk before and after prevalence-based filtering

Figure S2: Milk and infant fecal microbiome beta diversities and most abundant taxa

Figure S3: Differences in the alpha and beta diversities of milk and infant fecal community state types

Figure S4: HMO composition

Table S1: Selected cohort characteristics

Table S2: Milk microbial community state type (lactotype) characteristics

Table S3: Infant fecal microbial community state type (enterotype) characteristics

Table S4: Indicator taxa of the microbial lactotypes

Table S5: Indicator taxa of the infant microbial enterotypes

Table S6: HMO concentrations and ratios among microbial lactotypes

Table S7: HMO concentrations and ratios between enterotypes

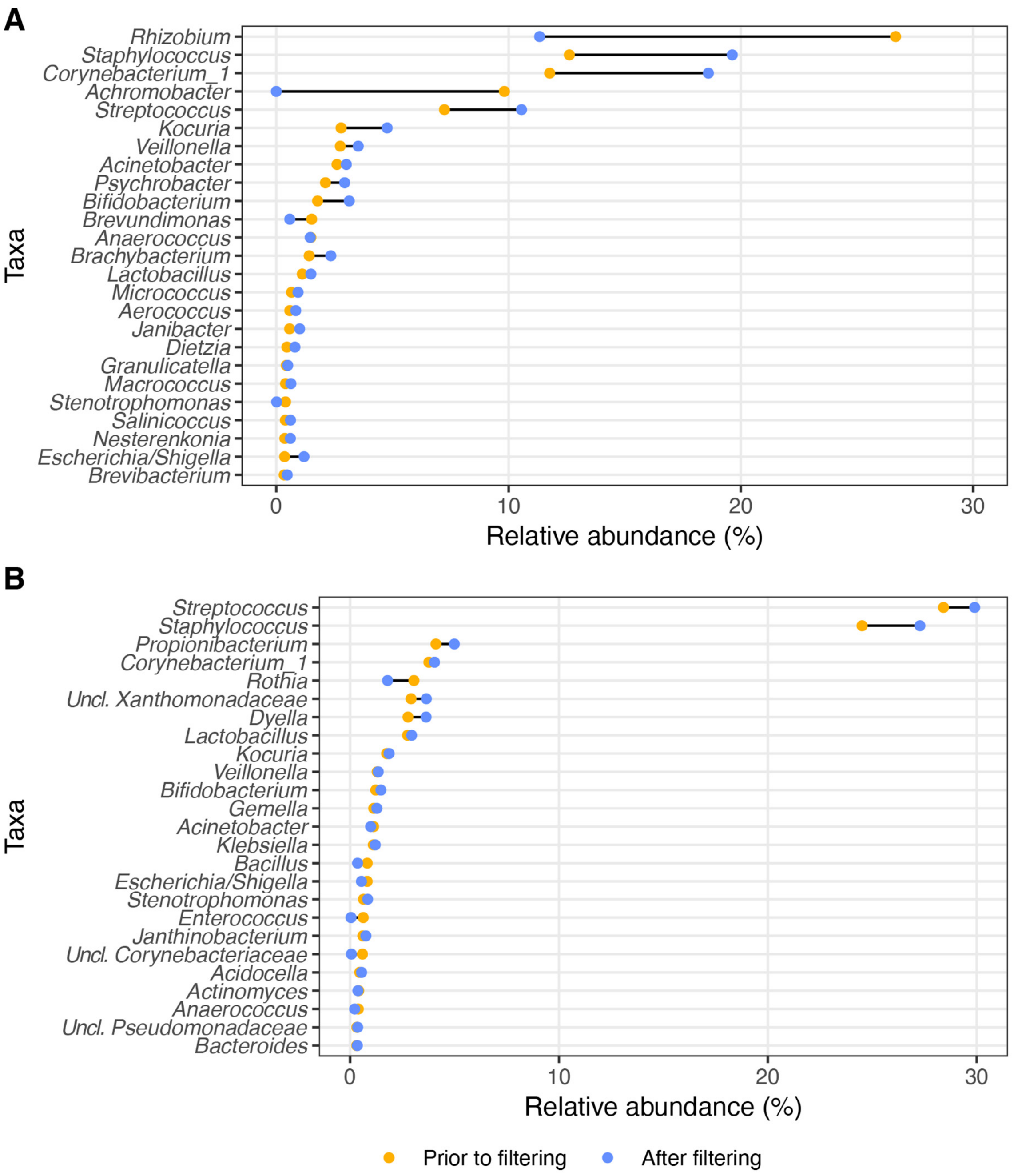

Figure S1. Relative abundances of the initial top 25 bacterial genera in milk before and after prevalence-based filtering**.** (**A**) Rural Ethiopia cohort and (**B**) all other**.** For the ETR milk samples, 580 of the initial 2,511 ASVs were validated to have statistical support as non-contaminants. For the remaining population cohort samples, 1,761 of the initial 8,964 ASVs were validated to have statistical support as non-contaminants. In general, there were marginal differences in the relative abundance of the most abundant genera (e.g., *Staphylococcus* and *Streptococcus*). Overall, most ASVs marked as contaminants in milk came from very low abundant genera (i.e., less than 0.001% across all milk samples). Only two of the initially most abundant taxa in milk were observed to have reductions in abundance > 10% (*Achromobacter* and *Rhizobium* in the ETR cohort). The reduction of *Rhizobium* abundance in the ETR cohort was attributed to the removal of a single *Rhizobium* ASV, although another abundant and prevalent *Rhizobium* ASV in the ETR samples not found in the respective negative controls was retained. Within the ETR samples the initial top 25 genera represented ~91% of all genera (from 458 genera) and in the decontam-filtered samples the top 25 genera represented ~92% of all genera (from 137 genera). Similarly, in the remaining population cohorts the initial top 25 genera represented ~86% of all genera (from 983 genera) and in the decontam-filtered samples the top 25 genera represented ~92% of all genera (from 334 genera).

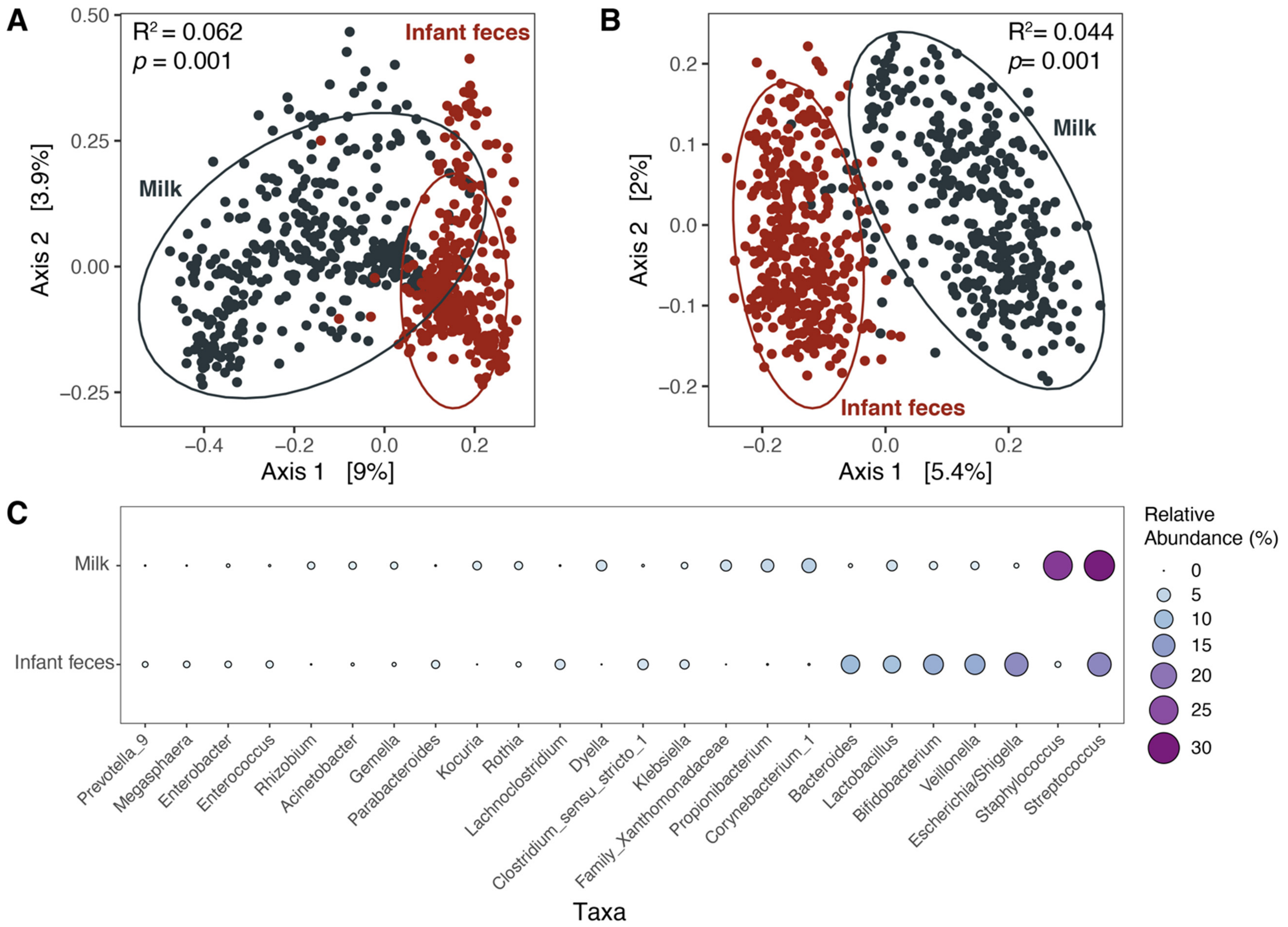

Figure S2. Milk and infant fecal microbiome beta diversities and most abundant taxa. (**A**) Microbial community structure (Bray-Curtis). (**B**) Microbial community membership (binary Jaccard). Differences between sample types were tested using PERMANOVA as implemented in vegan, with R^2 and p values listed. Ellipses represent the 95% confidence ellipses. (**C**) Bubble plot showing the most abundant taxa in milk and infant feces.

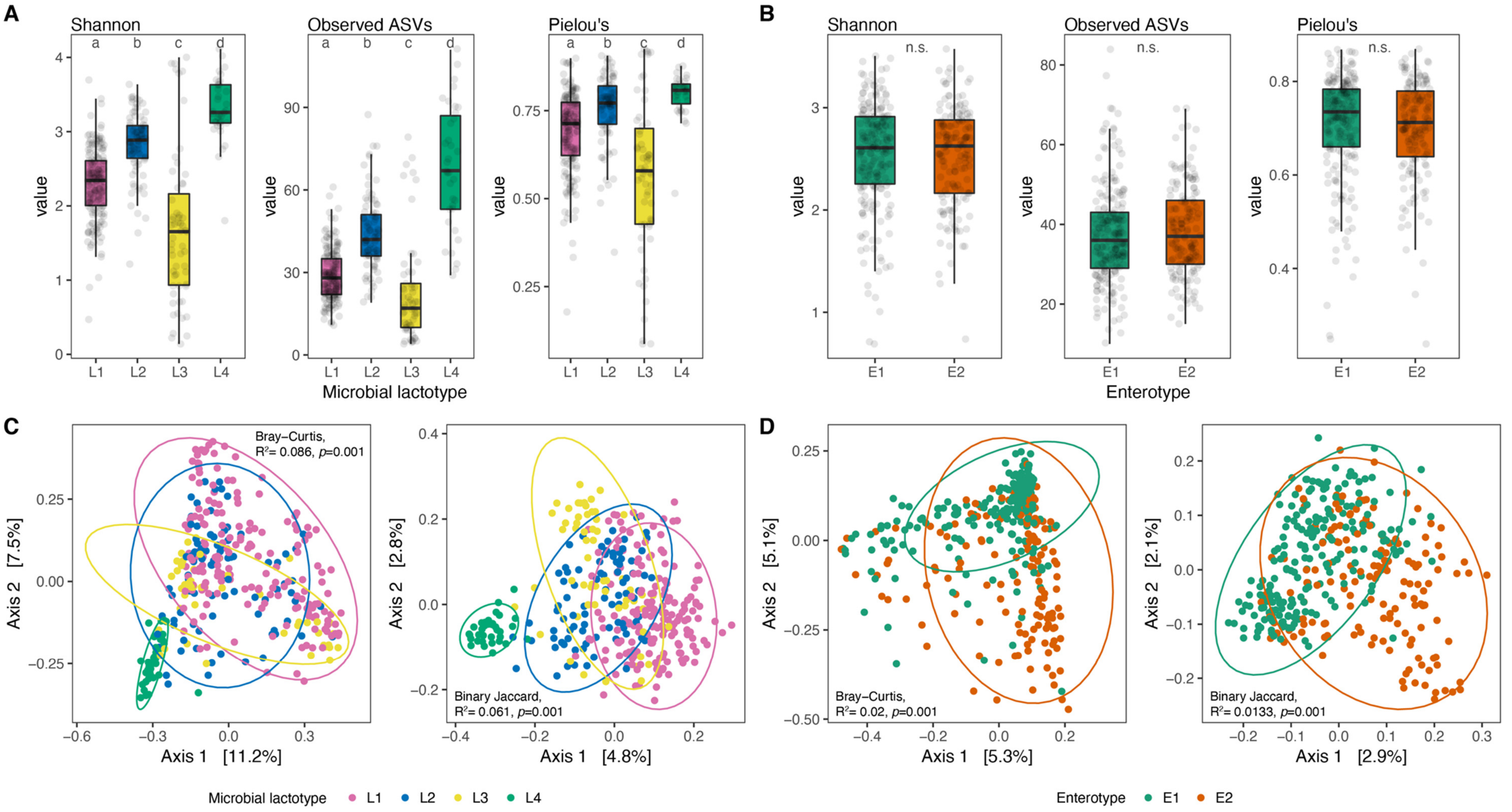

Figure S3. Differences in the alpha and beta diversities of milk and infant fecal community state types. The alpha and beta diversities of milk (**A**,**C**) and infant feces (**B**,**D**) were assessed and differences among and between community state types tested. Alpha diversity of milk (**A**) and infant feces was (**B**) assessed by computing Shannon diversity, observed ASVs, and Pielou's evenness metrics, and differences in these metrics was tested using the Kruskal-Wallis test, followed by Dunn's test (in milk samples) and the Mann-Whitney test (infant fecal). The beta diversities of milk (**C**) and infant feces (**D**) were assessed using Bray-Curtis and binary Jaccard metrics, with differences in community state types tested using PERMANOVA as implemented in vegan. Letters in panels **A** and **B** represent groups that statistically differ from one another ($p < 0.05$); n.s., not significant. Ellipses in panels **C** and **D** represent the 95% confidence ellipses.

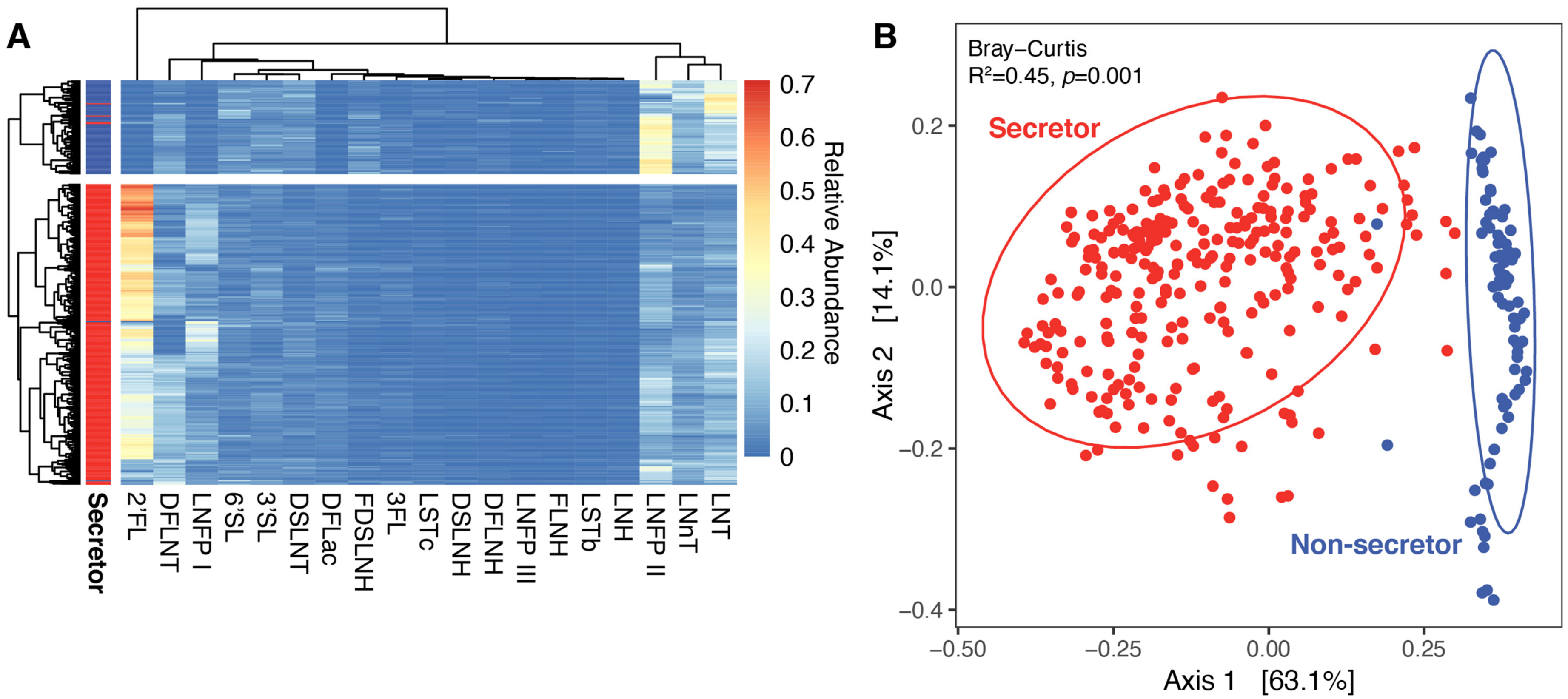

Figure S4. HMO composition. (**A**) Heatmap of the relative abundances of the 19 HMOs that represent the majority of HMOs present in milk. Maternal secretor status is denoted on the left-hand side (secretors, red; non-secretors, blue). Samples and HMOs are hierarchically clustered. (**B**) Multidimensional scaling (MDS) of HMO profiles based on Bray-Curtis distance. Samples are colored by maternal secretor status, and PERMANOVA R^2 and p values are given. Ellipses represent 95% confidence ellipses.

Table S1. Selected cohort characteristics

	Population												
Characteristic	All (n=357)	ETR (n=40)	ETU (n=26)	GBR (n=37)	GBU (n=36)	GN (n=28)	KE (n=41)	PE (n=42)	SP (n=36)	SW (n=21)	USC (n=12)	USW (n=38)	*p*
Maternal age (y)[1]	27.4±6.1	24.6±5.1	22.2±3.5	27.1±7.4	26.9±5.0	27.4±5.6	25.3±5.2	26.6±6.4	34.5±3.5	30.1±4.9	30.6±4.7	29.1±5.1	<0.001
Time postpartum (d)[2]	64.6±21.9	70.9±31.9	58.3±15.3	64.6±16.6	61.6±18.1	59±20.3	72.9±23.9	60.3±17	72.3±24.8	48.8±19.2	59.1±19.9	68.1±16	<0.001
Parity (n)	2.4±1.9	3.5±2.1	1.6±1.1	4.2±3.2	3.3±2	2.1±1	2.5±1.4	2±1.0	1.3±0.7	1.5±0.7	1.8±0.7	1.8±1.1	<0.001
Body mass index (kg/m^2)[3]	24.2±4.6	21.3±2.8	22.1±3.2	21.4±2.9	23.1±3.3	24.6±4.2	23.7±3.7	28±5.4	23.3±3.5	25.7±4.3	29±4.5	26.9±5.3	<0.001
Vaginal delivery, n (%)[4]	303 (86)	39 (100)	26 (100)	37 (100)	35 (97)	24 (89)	32 (80)	22 (52)	33 (92)	17 (81)	8 (67)	30 (79)	<0.001
Infant sex, Female, n (%)[5]	174 (49)	17 (44)	12 (46)	16 (44)	21 (58)	12 (43)	23 (56)	23 (55)	17 (47)	9 (43)	3 (27)	21 (55)	0.73
Infant weight-for-length z-score[6]	0.1±0.1	-0.3±1.0	-0.6±1.5	0.5±1.2	-0.1±1.1	-0.2±1.4	1.4±2.2	0.6±1.3	-0.3±1.1	-0.4±1.0	0.7±2.9	-1.0±1.1	<0.001
Exclusively breastfeeding, n (%)[7]	212 (60)	38 (95)	23 (89)	36 (97)	19 (53)	8 (29)	15 (37)	13 (31)	23 (64)	9 (43)	6 (50)	22 (60)	<0.001

Footnote: Categorical data are given as number of participants and, in parentheses, percent of total. Continuous data are provided as means ± standard deviation; *p* values were calculated from Kruskal-Wallis test for continuous data and Chi-squared test for categorical data. Infant weight-for-length z-scores were based on the 2006 WHO growth reference. [1], missing values for 3 dyads: 2 in GBR and 1 in GBU; [2], missing values for 1 dyad in GBR; [3], missing values for 7 dyads: 1 from GN, 2 from KE, 4 from USW; [4], missing values for 3 dyads: 1 in ETR, GN, and KE; [5], missing values for 3 dyads: 1 in ETR, GBR, and USC; [6], missing values for 10 dyads: 1 in ETR/GBR/GBU KE, 4 in USC, and 2 USW; [7], missing values for 1 dyad in USW.

Table S2. Milk microbial community state type (lactotype) characteristics

	Microbial Lactotype					
Characteristic	L1 (n=178)	L2 (n=85)	L3 (n=59)	L4 (n=35)	*p*	FDR *p*
Maternal age (y)[1]	28.2±5.8	26.1±6.7	28.9±5.9	24.2±4.4	<0.001	0.002
Time postpartum (d)[2]	61.8±20.5	69.1±19.8	62.1±18.8	71.8±33.6	0.030	0.045
Parity (n)	2.0±1.4	3.0±2.5	2.2±1.4	3.4±1.9	<0.001	0.002
Maternal body mass index (kg/m^2)[3]	25.5±4.8	22.8±4.2	23.9±3.9	21.3±2.9	<0.001	0.002
Maternal secretor status (secretor), n (%)	147 (83)	68 (80)	38 (64)	21 (60)	0.002	0.004
Vaginal delivery, n (%)[4]	146 (82)	73 (87)	50 (86)	34 (100)	0.053	0.060
Infant sex, Female, n (%)[5]	88 (50)	45 (54)	27 (46)	14 (41)	0.612	0.612
Infant weight-for-length z-score[6]	0.0±1.6	0.4±1.6	0.0±1.3	-0.3±1.1	0.053	0.060
Exclusively breastfeeding, n (%)[7]	99 (56)	54 (64)	25 (42)	34 (97)	<0.001	0.002

Footnote: Categorical data are given as number of participants and, in parentheses, percent of total. Continuous data are provided as means ± standard deviation; *p* values were calculated from Kruskal-Wallis test for continuous data and Chi-squared test for categorical data. Infant weight-for-length z-scores were based on the 2006 WHO growth reference. [1], missing values for 3 dyads: 2 in GBR and 1 in GBU; [2], missing values for 1 dyad in GBR; [3], missing values for 7 dyads: 1 from GN, 2 from KE, 4 from USW; [4], missing values for 3 dyads: 1 in ETR, GN, and KE; [5], missing values for 3 dyads: 1 in ETR, GBR, and USC; [6], missing values for 10 dyads: 1 in ETR/GBR/GBU KE, 4 in USC, and 2 USW; [7], missing values for 1 dyad in USW.

Table S3. Infant fecal microbial community state type (enterotype) characteristics				
	Microbial enterotype			
Characteristic	E1 (n=207)	E2 (n=150)	*p*	FDR *p*
Maternal age (y)[1]	28.1±6.2	26.6±5.9	0.022	0.040
Time postpartum (d)[2]	66±23.3	62±19.6	0.202	0.260
Parity (n)	2.2±1.7	2.8±2.0	<0.001	0.009
Maternal body mass index (kg/m^2)[3]	24.8±4.9	23.3±4.0	0.003	0.012
Maternal secretor status (secretor), n (%)	155 (75)	119 (79)	0.392	0.441
Vaginal delivery, n (%)[4]	170 (83)	132 (89)	0.182	0.260
Infant sex, Female, n (%)[5]	97 (48)	77 (51)	0.551	0.551
Infant weight-for-length z-score[6]	-0.1±1.7	0.2±1.4	0.004	0.012
Exclusively breastfeeding, n (%)[7]	112 (54)	102 (69)	0.009	0.020

Footnote: Categorical data are given as number of participants and, in parentheses, percent of total. Continuous data are provided as means ± standard deviation; *p* values were calculated from Mann-Whitney test for continuous data and Chi-squared test for categorical data. Infant weight-for-length z-scores were based on the 2006 WHO growth reference. [1], missing values for 3 dyads: 2 in GBR and 1 in GBU; [2], missing values for 1 dyad in GBR; [3], missing values for 7 dyads: 1 from GN, 2 from KE, 4 from USW; [4], missing values for 3 dyads: 1 in ETR, GN, and KE; [5], missing values for 3 dyads: 1 in ETR, GBR, and USC; [6], missing values for 10 dyads: 1 in ETR/GBR/GBU KE, 4 in USC, and 2 USW; [7], missing values for 1 dyad in USW.

**Table S4. Indicator taxa of the microbial lactotypes**					
Taxa	Microbial lactotype	indval	*p*	frequency	FDR *p*
Family Xanthomonadaceae	L1	0.54	0.001	235	0.006
Dyella	L1	0.48	0.001	242	0.006
Janthinobacterium	L1	0.41	0.001	185	0.006
Streptococcus	L1	0.41	0.001	348	0.006
Acidocella	L1	0.34	0.001	178	0.006
Flavobacterium	L1	0.22	0.001	73	0.006
Aeromonas	L1	0.34	0.002	144	0.011
Rothia	L1	0.28	0.003	155	0.015
Fluviicola	L1	0.09	0.006	20	0.028
Family Caulobacteraceae	L1	0.10	0.014	22	0.049
Family Clostridiaceae 1	L1	0.11	0.02	23	0.064
Ralstonia	L1	0.07	0.023	17	0.071
Yersinia	L1	0.20	0.025	104	0.075
Phylum Parcubacteria	L1	0.09	0.029	26	0.084
Burkholderia	L1	0.08	0.039	27	0.104
Fodinicola	L1	0.07	0.048	16	0.124
Hydrotalea	L1	0.07	0.058	19	0.142
Bradyrhizobium	L1	0.08	0.089	29	0.210
Morganella	L1	0.07	0.103	25	0.240
Clostridium sensu stricto 18	L1	0.07	0.128	21	0.283
Clostridium sensu stricto 9	L1	0.04	0.131	10	0.286
Family Bacillaceae	L1	0.05	0.134	15	0.286
hgcI clade	L1	0.03	0.134	6	0.286
Alpinimonas	L1	0.04	0.156	8	0.316
Sediminibacterium	L1	0.03	0.168	8	0.330
Cytophaga	L1	0.03	0.177	5	0.333
Acidovorax	L1	0.05	0.204	19	0.353
Limnohabitans	L1	0.05	0.221	18	0.377
Candidatus Flaviluna	L1	0.02	0.228	4	0.387
Achromobacter	L1	0.02	0.231	4	0.390
Acidibacter	L1	0.03	0.25	8	0.409
Phylum SM2F11	L1	0.02	0.263	4	0.424
Azorhizobium	L1	0.02	0.264	4	0.424
Family Microbacteriaceae	L1	0.02	0.264	4	0.424
Kingella	L1	0.02	0.276	4	0.439
Candidatus Rhodoluna	L1	0.03	0.282	13	0.445
Pseudarcicella	L1	0.03	0.312	12	0.484
Family Pseudomonadaceae	L1	0.09	0.326	69	0.504
Phylum Bacteroidetes	L1	0.03	0.329	7	0.506

Candidatus Planktoluna	L1	0.02	0.365	3	0.554
PRD01a011B	L1	0.02	0.392	3	0.587
Pantoea	L1	0.04	0.393	14	0.587
Schlegelella	L1	0.03	0.399	12	0.594
Phenylobacterium	L1	0.02	0.412	3	0.610
Phylum Proteobacteria	L1	0.02	0.431	3	0.628
Rhodococcus	L1	0.02	0.454	9	0.646
Rheinheimera	L1	0.02	0.462	4	0.650
Mesorhizobium	L1	0.02	0.466	8	0.652
Propionivibrio	L1	0.02	0.481	5	0.666
Candidatus Planktophila	L1	0.03	0.508	12	0.671
Order GR-WP33-30	L1	0.02	0.508	4	0.671
Family NS11-12 marine group	L1	0.02	0.51	4	0.671
Ewingella	L1	0.01	0.6	5	0.766
Cellulomonas	L1	0.01	0.607	2	0.766
Dechloromonas	L1	0.01	0.607	2	0.766
Nubsella	L1	0.01	0.607	2	0.766
Mangroviflexus	L1	0.01	0.611	2	0.766
Gemella	L1	0.18	0.612	163	0.766
Phylum TM6	L1	0.01	0.612	2	0.766
Marinomonas	L1	0.01	0.629	2	0.782
Family Comamonadaceae	L1	0.01	0.637	2	0.784
Helicobacter	L1	0.01	0.641	2	0.786
Gordonia	L1	0.01	0.646	2	0.789
Phylum Candidate division SR1	L1	0.01	0.649	4	0.789
Caulobacter	L1	0.01	0.65	2	0.789
Family Holophagaceae	L1	0.01	0.661	2	0.800
Family Rhodobiaceae	L1	0.01	0.678	3	0.815
Paludibacter	L1	0.01	0.691	2	0.821
Thauera	L1	0.01	0.691	2	0.821
Tolumonas	L1	0.01	0.696	3	0.823
Legionella	L1	0.01	0.712	2	0.839
Candidatus Aquiluna	L1	0.01	0.721	3	0.842
Romboutsia	L1	0.01	0.725	6	0.844
Clostridium sensu stricto 13	L1	0.01	0.734	3	0.850
Hyphomicrobium	L1	0.01	0.737	5	0.850
Sphingorhabdus	L1	0.01	0.742	3	0.852
Tepidimonas	L1	0.01	0.751	2	0.860
Lachnospiraceae UCG-001	L1	0.01	0.765	2	0.867
Haemophilus	L1	0.04	0.767	33	0.867
Pseudomonas	L1	0.10	0.784	93	0.881
Herbaspirillum	L1	0.01	0.836	6	0.922

Tepidiphilus	L1	0.01	0.866	3	0.947
Alcaligenes	L1	0.01	0.892	5	0.969
Scardovia	L1	0.01	0.924	4	0.998
Cloacibacterium	L1	0.01	0.951	12	1.000
Phylum WCHB1-60	L1	0.01	0.953	3	1.000
Azotobacter	L1	0.01	0.979	4	1.000
Bordetella	L1	0.01	1	1	1.000
Candidatus Alysiosphaera	L1	0.01	1	1	1.000
Class WCHB1-32	L1	0.01	1	1	1.000
Family Bacteroidales S24-7 group	L1	0.01	1	1	1.000
Family DSSF69	L1	0.01	1	1	1.000
Family Gemmatimonadaceae	L1	0.01	1	1	1.000
Geodermatophilus	L1	0.01	1	1	1.000
Pelosinus	L1	0.01	1	1	1.000
Serratia	L1	0.01	1	1	1.000
Sorangium	L1	0.01	1	1	1.000
Sulfuricurvum	L1	0.01	1	1	1.000
Family KCM-B-15	L1	0.01	1	2	1.000
Class Bacilli	L1	0.01	1	2	1.000
Family Paenibacillaceae	L1	0.00	1	2	1.000
Family Nocardioidaceae	L1	0.00	1	2	1.000
Paracocccus	L2	0.31	0.001	69	0.006
Microvirga	L2	0.14	0.001	13	0.006
Mobilicoccus	L2	0.13	0.001	11	0.006
Family Intrasporangiaceae	L2	0.18	0.002	21	0.011
Family Sphingomonadaceae	L2	0.12	0.002	11	0.011
Nocardioides	L2	0.15	0.003	22	0.015
Marmoricola	L2	0.09	0.003	10	0.015
Propionibacterium	L2	0.39	0.004	275	0.019
Saccharopolyspora	L2	0.09	0.006	10	0.028
Altererythrobacter	L2	0.06	0.007	5	0.032
Georgenia	L2	0.06	0.008	5	0.035
Piscicoccus	L2	0.09	0.011	15	0.041
Serinicoccus	L2	0.07	0.011	7	0.041
Order JG30-KF-CM45	L2	0.05	0.013	6	0.046
Turicibacter	L2	0.07	0.015	11	0.050
Propioniciclava	L2	0.06	0.023	11	0.071
Massilia	L2	0.06	0.023	8	0.071
Atopobium	L2	0.13	0.025	48	0.075
Thermomonas	L2	0.05	0.026	4	0.077
Family Planococcaceae	L2	0.04	0.027	3	0.080
Rubellimicrobium	L2	0.05	0.028	4	0.082

Family Rhodobacteraceae	L2	0.05	0.032	8	0.090
Streptomyces	L2	0.05	0.033	8	0.092
Sporosarcina	L2	0.04	0.036	3	0.098
Actinomyces	L2	0.15	0.042	82	0.111
Family FamilyI	L2	0.05	0.045	4	0.118
Blastococcus	L2	0.05	0.046	4	0.120
Agrococcus	L2	0.05	0.048	4	0.124
Aureimonas	L2	0.04	0.049	3	0.124
Lysobacter	L2	0.04	0.049	3	0.124
Aquitalea	L2	0.05	0.05	10	0.126
Tessaracoccus	L2	0.05	0.051	11	0.128
Actinopolyspora	L2	0.03	0.062	4	0.150
Peptoniphilus	L2	0.07	0.102	24	0.239
Empedobacter	L2	0.05	0.122	14	0.275
Singulisphaera	L2	0.03	0.124	6	0.278
Yimella	L2	0.02	0.131	2	0.286
Sphingomonas	L2	0.05	0.133	17	0.286
Pandoraea	L2	0.02	0.133	2	0.286
Phycicoccus	L2	0.02	0.137	2	0.291
Flavisolibacter	L2	0.02	0.138	2	0.291
Erythrobacter	L2	0.02	0.141	2	0.294
Nitrosomonas	L2	0.02	0.141	2	0.294
Pontibacter	L2	0.03	0.145	6	0.299
Class TA18	L2	0.02	0.145	2	0.299
Pedobacter	L2	0.06	0.146	20	0.299
Bdellovibrio	L2	0.03	0.156	8	0.316
Class Thermomicrobia	L2	0.02	0.159	2	0.321
Mycobacterium	L2	0.02	0.166	2	0.330
Phylum Gracilibacteria	L2	0.02	0.168	4	0.330
Class Thermoleophilia	L2	0.02	0.169	2	0.330
Nocardiopsis	L2	0.02	0.17	2	0.330
Salinimicrobium	L2	0.02	0.17	2	0.330
Acidiphilium	L2	0.02	0.171	2	0.330
Bhargavaea	L2	0.02	0.171	2	0.330
Ornithinicoccus	L2	0.02	0.173	2	0.332
Family Micrococcaceae	L2	0.02	0.176	2	0.333
Peptostreptococcus	L2	0.05	0.178	13	0.333
Psychrobacillus	L2	0.02	0.178	2	0.333
Defluviicoccus	L2	0.02	0.18	2	0.334
Isoptericola	L2	0.02	0.181	2	0.334
Family 0319-6M6	L2	0.02	0.182	2	0.334
Gaiella	L2	0.02	0.182	2	0.334

Mogibacterium	L2	0.02	0.183	2	0.334
Filibacter	L2	0.02	0.185	2	0.334
Family Micromonosporaceae	L2	0.02	0.187	2	0.336
Tetrasphaera	L2	0.04	0.189	11	0.338
Citricoccus	L2	0.02	0.193	2	0.342
Class Gitt-GS-136	L2	0.02	0.195	2	0.344
Cellulosilyticum	L2	0.02	0.2	2	0.351
Order Sphingobacteriales	L2	0.02	0.208	2	0.358
Paenibacillus	L2	0.02	0.237	4	0.396
Variovorax	L2	0.06	0.24	30	0.398
Anoxybacillus	L2	0.02	0.267	4	0.427
Order Obscuribacterales	L2	0.03	0.295	8	0.462
Family Cryomorphaceae	L2	0.01	0.428	2	0.626
Aggregatibacter	L2	0.02	0.434	4	0.630
Micromonospora	L2	0.01	0.445	2	0.641
Family MN 122.2a	L2	0.01	0.457	2	0.648
Ezakiella	L2	0.01	0.46	3	0.650
Noviherbaspirillum	L2	0.01	0.467	2	0.652
Family JG34-KF-161	L2	0.01	0.486	1	0.671
Ensifer	L2	0.01	0.492	1	0.671
Dermacoccus	L2	0.01	0.496	1	0.671
Xanthomonas	L2	0.01	0.499	1	0.671
Bryocella	L2	0.01	0.499	2	0.671
Propionimicrobium	L2	0.01	0.502	1	0.671
Order Ardenticatenales	L2	0.01	0.506	1	0.671
Sanguibacter	L2	0.01	0.511	1	0.671
Family MIZ14	L2	0.01	0.512	1	0.671
Family Rhodothermaceae	L2	0.01	0.512	1	0.671
Yaniella	L2	0.01	0.522	1	0.682
Finegoldia	L2	0.05	0.533	25	0.692
Roseomonas	L2	0.02	0.59	8	0.757
Family Flavobacteriaceae	L2	0.02	0.615	10	0.767
Alicycliphilus	L2	0.02	0.635	7	0.784
Halomonas	L2	0.03	0.637	20	0.784
Sphaerotilus	L2	0.01	0.676	4	0.815
Oxalophagus	L2	0.01	0.692	4	0.821
Citrobacter	L2	0.02	0.718	15	0.841
Dolosigranulum	L2	0.02	0.738	14	0.850
Undibacterium	L2	0.01	0.757	2	0.864
Collinsella	L2	0.01	0.779	2	0.878
Order PeM15	L2	0.01	0.788	4	0.882
Megamonas	L2	0.01	0.79	3	0.882

Aquamicrobium	L2	0.01	0.804	2	0.895
Family Bradyrhizobiaceae	L2	0.01	0.873	2	0.952
Microvirgula	L2	0.01	0.988	8	1.000
Haematobacter	L2	0.01	0.994	5	1.000
Solitalea	L2	0.01	1	2	1.000
Family 480-2	L2	0.01	1	3	1.000
Alishewanella	L2	0.01	1	3	1.000
Roseateles	L2	0.01	1	3	1.000
Oribacterium	L2	0.01	1	2	1.000
Dialister	L2	0.00	1	2	1.000
Rhizobacter	L2	0.00	1	2	1.000
Klebsiella	L3	0.40	0.001	36	0.006
Lactobacillus	L3	0.39	0.001	150	0.006
Escherichia/Shigella	L3	0.29	0.001	47	0.006
Bacteroides	L3	0.22	0.001	27	0.006
Campylobacter	L3	0.17	0.001	13	0.006
Parabacteroides	L3	0.12	0.001	13	0.006
Paucisalibacillus	L3	0.08	0.004	6	0.019
Sphingobium	L3	0.07	0.01	9	0.041
Ochrobactrum	L3	0.06	0.013	7	0.046
Gluconobacter	L3	0.08	0.015	7	0.050
Order Clostridiales	L3	0.05	0.015	3	0.050
Family Enterobacteriaceae	L3	0.11	0.017	31	0.055
Family Rhizobiaceae	L3	0.03	0.023	2	0.071
Akkermansia	L3	0.05	0.024	3	0.073
Tyzzerella 3	L3	0.03	0.03	2	0.086
Tatumella	L3	0.07	0.031	9	0.088
Proteus	L3	0.03	0.035	2	0.097
Megasphaera	L3	0.05	0.036	4	0.098
Family Lachnospiraceae	L3	0.03	0.037	2	0.099
Prevotella	L3	0.05	0.11	13	0.253
Order Sphingomonadales	L3	0.03	0.126	4	0.281
Lachnoclostridium	L3	0.04	0.191	9	0.340
Enterobacter	L3	0.14	0.203	75	0.353
Solobacterium	L3	0.03	0.216	5	0.370
Nostoc	L3	0.02	0.235	1	0.395
Porphyrobacter	L3	0.02	0.238	1	0.396
Capnocytophaga	L3	0.02	0.253	2	0.412
Family Saprospiraceae	L3	0.02	0.277	1	0.439
Negativicoccus	L3	0.02	0.284	2	0.446
Filifactor	L3	0.02	0.309	2	0.481
Weissella	L3	0.05	0.353	29	0.541

Holdemanella	L3	0.01	0.365	2	0.554
Eubacterium	L3	0.01	0.369	2	0.556
Phylum SHA-109	L3	0.01	0.416	2	0.614
Order Verrucomicrobiales	L3	0.01	0.428	2	0.626
Stenotrophomonas	L3	0.15	0.441	97	0.638
Prevotella 9	L3	0.02	0.453	5	0.646
Parvimonas	L3	0.02	0.469	3	0.652
Fusobacterium	L3	0.02	0.534	10	0.692
Clostridium sensu stricto 1	L3	0.04	0.54	32	0.698
Actinobacillus	L3	0.01	0.688	3	0.821
Staphylococcus	L3	0.27	0.714	352	0.839
Pelomonas	L3	0.01	0.83	5	0.919
Family Anaerolineaceae	L3	0.01	0.848	2	0.933
Polynucleobacter	L3	0.01	0.866	5	0.947
Bacillus	L3	0.06	0.9	47	0.975
Macrococcus	L4	0.81	0.001	38	0.006
Brachybacterium	L4	0.80	0.001	67	0.006
Aerococcus	L4	0.68	0.001	35	0.006
Janibacter	L4	0.66	0.001	48	0.006
Psychrobacter	L4	0.65	0.001	45	0.006
Nesterenkonia	L4	0.62	0.001	36	0.006
Corynebacterium 1	L4	0.60	0.001	259	0.006
Arthrobacter	L4	0.57	0.001	38	0.006
Rhizobium	L4	0.56	0.001	37	0.006
Brevibacterium	L4	0.56	0.001	46	0.006
Facklamia	L4	0.53	0.001	27	0.006
Anaerococcus	L4	0.51	0.001	76	0.006
Kocuria	L4	0.51	0.001	157	0.006
Salinicoccus	L4	0.47	0.001	23	0.006
Jeotgalicoccus	L4	0.46	0.001	16	0.006
Micrococcus	L4	0.42	0.001	97	0.006
Veillonella	L4	0.38	0.001	191	0.006
Bifidobacterium	L4	0.34	0.001	172	0.006
Delftia	L4	0.33	0.001	29	0.006
Family Staphylococcaceae	L4	0.31	0.001	11	0.006
Ornithinimicrobium	L4	0.31	0.001	53	0.006
Microbacterium	L4	0.31	0.001	60	0.006
Granulicatella	L4	0.31	0.001	72	0.006
Kytococcus	L4	0.30	0.001	13	0.006
Lactococcus	L4	0.30	0.001	14	0.006
Globicatella	L4	0.30	0.001	13	0.006
Brevundimonas	L4	0.29	0.001	45	0.006

Dietzia	L4	0.29	0.001	65	0.006
Leuconostoc	L4	0.28	0.001	30	0.006
Phylum Saccharibacteria	L4	0.27	0.001	38	0.006
Olivibacter	L4	0.20	0.001	7	0.006
Planomicrobium	L4	0.20	0.001	7	0.006
Sphingobacterium	L4	0.17	0.001	17	0.006
Nosocomiicoccus	L4	0.17	0.001	6	0.006
Pediococcus	L4	0.17	0.001	6	0.006
Enterococcus	L4	0.15	0.001	24	0.006
Alkalibacterium	L4	0.14	0.001	8	0.006
Family Carnobacteriaceae	L4	0.14	0.001	5	0.006
Solibacillus	L4	0.14	0.001	5	0.006
Raoultella	L4	0.12	0.001	9	0.006
Aliicoccus	L4	0.11	0.001	4	0.006
Order Micrococcales	L4	0.11	0.001	6	0.006
Demequina	L4	0.09	0.001	3	0.006
Duganella	L4	0.09	0.001	3	0.006
Deinococcus	L4	0.15	0.002	17	0.011
Acetobacter	L4	0.12	0.002	8	0.011
Porphyromonas	L4	0.11	0.002	16	0.011
Clostridium sensu stricto 12	L4	0.10	0.002	6	0.011
Luteimonas	L4	0.10	0.003	10	0.015
Rahnella	L4	0.09	0.003	3	0.015
Comamonas	L4	0.12	0.004	22	0.019
Lautropia	L4	0.08	0.004	5	0.019
Promicromonospora	L4	0.06	0.006	2	0.028
Leifsonia	L4	0.06	0.008	2	0.035
Zhihengliuella	L4	0.06	0.008	2	0.035
Exiguobacterium	L4	0.07	0.009	7	0.039
Dysgonomonas	L4	0.06	0.01	2	0.041
Family Brucellaceae	L4	0.06	0.01	2	0.041
Paenalcaligenes	L4	0.06	0.01	2	0.041
Pseudoalteromonas	L4	0.06	0.01	2	0.041
Pseudobutyrivibrio	L4	0.06	0.01	2	0.041
Family Aerococcaceae	L4	0.06	0.011	2	0.041
Family Dermabacteraceae	L4	0.06	0.011	2	0.041
Fastidiosipila	L4	0.06	0.011	2	0.041
Oceanitalea	L4	0.06	0.011	2	0.041
Salana	L4	0.06	0.011	2	0.041
Curtobacterium	L4	0.05	0.011	3	0.041
Marinococcus	L4	0.06	0.012	2	0.044
Marinilactibacillus	L4	0.06	0.013	2	0.046

Planococcus	L4	0.06	0.013	2	0.046
Tropicimonas	L4	0.06	0.013	2	0.046
Actinotalea	L4	0.06	0.015	2	0.050
Neisseria	L4	0.11	0.016	35	0.053
Methylobacterium	L4	0.08	0.016	17	0.053
Peptoclostridium	L4	0.07	0.017	11	0.055
Flaviflexus	L4	0.06	0.017	2	0.055
Chryseomicrobium	L4	0.06	0.029	12	0.084
Erysipelatoclostridium	L4	0.05	0.037	7	0.099
Alloprevotella	L4	0.08	0.053	22	0.132
Aquabacterium	L4	0.04	0.055	4	0.136
Knoellia	L4	0.04	0.061	8	0.149
Leptotrichia	L4	0.04	0.067	5	0.161
Atopostipes	L4	0.03	0.071	4	0.170
Acinetobacter	L4	0.26	0.077	156	0.183
Gardnerella	L4	0.06	0.107	20	0.247
Ignavigranum	L4	0.03	0.111	4	0.253
Bergeyella	L4	0.07	0.114	22	0.259
Prevotella 7	L4	0.06	0.178	26	0.333
Agromyces	L4	0.03	0.184	5	0.334
Moraxella	L4	0.12	0.203	66	0.353
Pseudonocardia	L4	0.02	0.245	3	0.404
Devosia	L4	0.02	0.25	6	0.409
Corynebacterium	L4	0.04	0.366	15	0.554
Family Neisseriaceae	L4	0.05	0.452	33	0.646
Intestinibacter	L4	0.04	0.504	26	0.671
Abiotrophia	L4	0.02	0.506	5	0.671
Family Corynebacteriaceae	L4	0.03	0.553	17	0.712
Chryseobacterium	L4	0.11	0.76	72	0.865
Family Halomonadaceae	L4	0.01	0.818	7	0.908

Table S5. Indicator taxa of the infant microbial enterotypes					
Taxa	Microbial enterotype	indval	*p*	frequency	FDR *p*
Bacteroides	E1	0.82	0.001	262	0.017
Lachnoclostridium	E1	0.56	0.001	148	0.017
Clostridium sensu stricto 1	E1	0.44	0.001	128	0.017
Parabacteroides	E1	0.32	0.001	88	0.017
Erysipelatoclostridium	E1	0.27	0.001	68	0.017
Enterococcus	E1	0.45	0.002	188	0.023
Family Enterobacteriaceae	E1	0.20	0.002	67	0.023
Citrobacter	E1	0.16	0.002	38	0.023
Peptoclostridium	E1	0.15	0.002	35	0.023
Ruminiclostridium 5	E1	0.10	0.002	26	0.023
Erysipelotrichaceae Incertae Sedis	E1	0.09	0.002	20	0.023
Enterobacter	E1	0.24	0.003	77	0.031
Escherichia/Shigella	E1	0.56	0.004	329	0.039
Flavonifractor	E1	0.09	0.005	23	0.045
Alistipes	E1	0.06	0.006	13	0.053
Blautia	E1	0.11	0.009	26	0.072
Dorea	E1	0.07	0.011	17	0.085
Phascolarctobacterium	E1	0.05	0.015	10	0.113
Haemophilus	E1	0.17	0.022	62	0.162
Family Ruminococcaceae	E1	0.06	0.024	16	0.172
Hungatella	E1	0.04	0.031	8	0.216
Raoultella	E1	0.09	0.04	31	0.259
Anaeroglobus	E1	0.03	0.04	7	0.259
Tyzzerella 4	E1	0.04	0.053	10	0.328
Faecalibacterium	E1	0.03	0.066	6	0.396
Negativicoccus	E1	0.11	0.108	46	0.554
Anaerostipes	E1	0.03	0.108	8	0.554
Morganella	E1	0.04	0.115	11	0.579
Bilophila	E1	0.03	0.124	10	0.613
Varibaculum	E1	0.04	0.133	11	0.646
Akkermansia	E1	0.06	0.139	19	0.652
Dysgonomonas	E1	0.05	0.139	14	0.652
Sutterella	E1	0.10	0.143	35	0.659
Succinivibrio	E1	0.02	0.185	4	0.733
Butyricimonas	E1	0.02	0.186	4	0.733
Megasphaera	E1	0.13	0.193	64	0.739
Lactococcus	E1	0.06	0.193	23	0.739
Eggerthella	E1	0.04	0.207	17	0.782
Terrisporobacter	E1	0.01	0.231	3	0.861

Subdoligranulum	E1	0.01	0.236	3	0.867
Catabacter	E1	0.01	0.25	3	0.894
[Eubacterium] hallii group	E1	0.01	0.255	3	0.894
Family Bacteroidales S24-7 group	E1	0.01	0.261	3	0.894
Parascardovia	E1	0.01	0.262	3	0.894
Epulopiscium	E1	0.01	0.263	3	0.894
Eubacterium	E1	0.04	0.347	15	0.993
Intestinibacter	E1	0.03	0.355	15	0.993
[Eubacterium] coprostanoligenes group	E1	0.01	0.494	2	0.993
Lachnospiraceae Incertae Sedis	E1	0.01	0.494	2	0.993
Microbacterium	E1	0.01	0.502	2	0.993
Anaerotruncus	E1	0.01	0.504	2	0.993
Barnesiella	E1	0.01	0.507	2	0.993
Gordonibacter	E1	0.01	0.507	2	0.993
Aeromonas	E1	0.01	0.51	2	0.993
Ruminococcaceae UCG-014	E1	0.01	0.513	2	0.993
Christensenellaceae R-7 group	E1	0.01	0.515	2	0.993
Providencia	E1	0.01	0.517	2	0.993
Acidovorax	E1	0.01	0.521	2	0.993
Comamonas	E1	0.01	0.521	2	0.993
Hyphomicrobium	E1	0.01	0.521	2	0.993
Salmonella	E1	0.01	0.522	2	0.993
Syntrophococcus	E1	0.01	0.535	2	1.000
Odoribacter	E1	0.01	0.553	5	1.000
Collinsella	E1	0.02	0.568	9	1.000
Moraxella	E1	0.01	0.612	3	1.000
Lachnospiraceae UCG-004	E1	0.01	0.693	7	1.000
Propionibacterium	E1	0.05	0.699	26	1.000
Leuconostoc	E1	0.03	0.699	18	1.000
Parasutterella	E1	0.01	0.706	4	1.000
Prevotella 9	E1	0.03	0.708	17	1.000
Acidocella	E1	0.01	0.742	3	1.000
Scardovia	E1	0.01	0.747	6	1.000
Fusobacterium	E1	0.04	0.754	22	1.000
Acinetobacter	E1	0.04	0.755	21	1.000
Catenibacterium	E1	0.02	0.765	7	1.000
Clostridium sensu stricto 13	E1	0.01	0.765	3	1.000
Aerococcus	E1	0.01	0.772	5	1.000
Trabulsiella	E1	0.01	0.81	6	1.000
Family Clostridiaceae 1	E1	0.01	0.856	3	1.000
Paucisalibacillus	E1	0.01	0.857	5	1.000
Actinobacillus	E1	0.00	0.858	3	1.000

Lactonifactor	E1	0.01	0.879	4	1.000
Finegoldia	E1	0.07	0.884	38	1.000
Solobacterium	E1	0.01	0.925	5	1.000
Family Bifidobacteriaceae	E1	0.01	0.933	4	1.000
Peptoniphilus	E1	0.06	0.958	43	1.000
Anaerococcus	E1	0.05	0.959	37	1.000
Kluyvera	E1	0.02	0.973	12	1.000
Aerosphaera	E1	0.00	1	1	1.000
Aggregatibacter	E1	0.00	1	1	1.000
Ameyamaea	E1	0.00	1	1	1.000
Anaerosalibacter	E1	0.00	1	1	1.000
Anaerosporobacter	E1	0.00	1	1	1.000
Aquabacterium	E1	0.00	1	1	1.000
Bradyrhizobium	E1	0.00	1	1	1.000
Caulobacter	E1	0.00	1	1	1.000
Class Actinobacteria	E1	0.00	1	1	1.000
Class Alphaproteobacteria	E1	0.00	1	1	1.000
Class Mollicutes	E1	0.00	1	1	1.000
Clostridium sensu stricto 18	E1	0.00	1	1	1.000
Clostridium sensu stricto 2	E1	0.00	1	1	1.000
Clostridium sensu stricto 9	E1	0.00	1	1	1.000
Coprobacter	E1	0.00	1	1	1.000
Devosia	E1	0.00	1	1	1.000
Dyella	E1	0.00	1	1	1.000
Erysipelotrichaceae UCG-006	E1	0.00	1	1	1.000
Facklamia	E1	0.00	1	1	1.000
Family 11-24	E1	0.00	1	1	1.000
Family Bacteroidales RF16 group	E1	0.00	1	1	1.000
Family Enterococcaceae	E1	0.00	1	1	1.000
Family Hyphomicrobiaceae	E1	0.00	1	1	1.000
Family Leptotrichiaceae	E1	0.00	1	1	1.000
Family Planococcaceae	E1	0.00	1	1	1.000
Family Pseudomonadaceae	E1	0.00	1	1	1.000
Family Rhodobacteraceae	E1	0.00	1	1	1.000
Family Sphingomonadaceae	E1	0.00	1	1	1.000
Flavisolibacter	E1	0.00	1	1	1.000
Geobacillus	E1	0.00	1	1	1.000
Gluconobacter	E1	0.00	1	1	1.000
Helicobacter	E1	0.00	1	1	1.000
Intestinimonas	E1	0.00	1	1	1.000
Johnsonella	E1	0.00	1	1	1.000
Kingella	E1	0.00	1	1	1.000

Kocuria	E1	0.00	1	1	1.000
Lachnoclostridium 5	E1	0.00	1	1	1.000
Leptotrichia	E1	0.00	1	1	1.000
Massilia	E1	0.00	1	1	1.000
Megamonas	E1	0.00	1	1	1.000
Novosphingobium	E1	0.00	1	1	1.000
Order Gastranaerophilales	E1	0.00	1	1	1.000
Order Micrococcales	E1	0.00	1	1	1.000
Order Mollicutes RF9	E1	0.00	1	1	1.000
Oribacterium	E1	0.00	1	1	1.000
Paludibacter	E1	0.00	1	1	1.000
Paraprevotella	E1	0.00	1	1	1.000
Patulibacter	E1	0.00	1	1	1.000
Pediococcus	E1	0.00	1	1	1.000
Phylum Bacteroidetes	E1	0.00	1	1	1.000
Phylum SM2F11	E1	0.00	1	1	1.000
Phylum TM6	E1	0.00	1	1	1.000
Pigmentiphaga	E1	0.00	1	1	1.000
Polynucleobacter	E1	0.00	1	1	1.000
Prevotellaceae NK3B31 group	E1	0.00	1	1	1.000
Pseudoclavibacter	E1	0.00	1	1	1.000
Roseomonas	E1	0.00	1	1	1.000
Ruminiclostridium	E1	0.00	1	1	1.000
Ruminococcaceae UCG-004	E1	0.00	1	1	1.000
Ruminococcaceae UCG-005	E1	0.00	1	1	1.000
Sarcina	E1	0.00	1	1	1.000
Selenomonas 3	E1	0.00	1	1	1.000
Streptomyces	E1	0.00	1	1	1.000
Tepidimicrobium	E1	0.00	1	1	1.000
Thalassospira	E1	0.00	1	1	1.000
Trueperella	E1	0.00	1	1	1.000
Tyzzerella	E1	0.00	1	1	1.000
[Ruminococcus] gauvreauii group	E1	0.00	1	2	1.000
Brevibacterium	E1	0.00	1	2	1.000
Brevundimonas	E1	0.00	1	2	1.000
Mogibacterium	E1	0.00	1	4	1.000
Rhizobium	E1	0.00	1	2	1.000
[Eubacterium] nodatum group	E1	0.00	1	3	1.000
Alloscardovia	E1	0.00	1	2	1.000
Family Prevotellaceae	E1	0.00	1	4	1.000
Family Methylophilaceae	E1	0.00	1	2	1.000
Murdochiella	E1	0.00	1	2	1.000

Bacillus	E1	0.00	1	2	1.000
Macrococcus	E1	0.00	1	2	1.000
Fusicatenibacter	E1	0.00	1	2	1.000
Desulfovibrio	E1	0.00	1	2	1.000
Streptococcus	E2	0.81	0.001	352	0.017
Bifidobacterium	E2	0.66	0.001	318	0.017
Lactobacillus	E2	0.63	0.001	295	0.017
Gemella	E2	0.60	0.001	138	0.017
Rothia	E2	0.53	0.001	135	0.017
Staphylococcus	E2	0.52	0.001	223	0.017
Atopobium	E2	0.31	0.001	68	0.017
Granulicatella	E2	0.30	0.001	59	0.017
Prevotella	E2	0.27	0.001	75	0.017
Tyzzerella 3	E2	0.22	0.001	34	0.017
Order Lactobacillales	E2	0.09	0.001	13	0.017
Corynebacterium 1	E2	0.21	0.002	72	0.023
Gardnerella	E2	0.06	0.002	10	0.023
Actinomyces	E2	0.28	0.003	117	0.031
Porphyromonas	E2	0.10	0.004	24	0.039
Dolosigranulum	E2	0.05	0.005	9	0.045
Lachnoanaerobaculum	E2	0.04	0.007	9	0.060
Peptostreptococcus	E2	0.11	0.009	29	0.072
Fastidiosipila	E2	0.03	0.04	4	0.259
Ezakiella	E2	0.08	0.044	25	0.278
Candidatus Rhodoluna	E2	0.02	0.067	3	0.396
Phylum Firmicutes	E2	0.02	0.07	3	0.402
Family Coriobacteriaceae	E2	0.03	0.071	7	0.402
Mitsuokella	E2	0.02	0.077	3	0.427
Class Bacilli	E2	0.03	0.085	6	0.462
Prevotella 6	E2	0.04	0.091	12	0.485
Flavobacterium	E2	0.03	0.148	8	0.671
Pseudomonas	E2	0.02	0.156	5	0.696
Thermomonas	E2	0.01	0.167	2	0.709
Holdemanella	E2	0.06	0.168	22	0.709
Weissella	E2	0.06	0.171	19	0.709
Paracocccus	E2	0.01	0.172	2	0.709
Sphingomonas	E2	0.01	0.172	2	0.709
Sphingobacterium	E2	0.01	0.18	2	0.731
Olsenella	E2	0.02	0.245	7	0.889
Senegalimassilia	E2	0.02	0.28	5	0.940
Family Flavobacteriaceae	E2	0.01	0.308	4	0.993
Psychrobacter	E2	0.01	0.311	4	0.993

Proteus	E2	0.04	0.352	16	0.993
Allisonella	E2	0.01	0.355	3	0.993
Klebsiella	E2	0.19	0.374	114	0.993
Stenotrophomonas	E2	0.03	0.384	14	0.993
Sneathia	E2	0.01	0.395	1	0.993
Bdellovibrio	E2	0.01	0.4	1	0.993
Rhodococcus	E2	0.01	0.407	1	0.993
Abiotrophia	E2	0.01	0.408	1	0.993
Ottowia	E2	0.01	0.409	1	0.993
Taibaiella	E2	0.01	0.409	1	0.993
Fluviicola	E2	0.01	0.411	1	0.993
Sphingopyxis	E2	0.01	0.411	1	0.993
Limnohabitans	E2	0.01	0.417	1	0.993
Candidatus Planktophila	E2	0.01	0.418	1	0.993
Olivibacter	E2	0.01	0.418	1	0.993
Parapedobacter	E2	0.01	0.418	1	0.993
[Eubacterium] brachy group	E2	0.01	0.419	1	0.993
Methylobacterium	E2	0.01	0.42	1	0.993
Family Family XIII	E2	0.01	0.426	1	0.993
Haloferula	E2	0.01	0.426	1	0.993
Ochrobactrum	E2	0.01	0.426	1	0.993
Azorhizobium	E2	0.01	0.427	1	0.993
Pseudacidovorax	E2	0.01	0.427	1	0.993
Kurthia	E2	0.01	0.428	1	0.993
Achromobacter	E2	0.01	0.429	1	0.993
Brachybacterium	E2	0.01	0.429	1	0.993
Family Microbacteriaceae	E2	0.01	0.43	1	0.993
Robinsoniella	E2	0.01	0.43	1	0.993
Cruoricaptor	E2	0.01	0.432	1	0.993
Class Clostridia	E2	0.01	0.433	1	0.993
Family Peptostreptococcaceae	E2	0.02	0.438	5	0.993
Niabella	E2	0.01	0.439	1	0.993
Weeksella	E2	0.01	0.439	1	0.993
Bordetella	E2	0.01	0.441	1	0.993
Rhodopseudomonas	E2	0.01	0.441	1	0.993
Arthrobacter	E2	0.01	0.443	1	0.993
Class WCHB1-32	E2	0.01	0.451	1	0.993
Romboutsia	E2	0.01	0.456	1	0.993
Hafnia	E2	0.01	0.468	3	0.993
Prevotella 7	E2	0.02	0.469	6	0.993
Class Gammaproteobacteria	E2	0.01	0.477	3	0.993
Order Bacteroidales	E2	0.02	0.481	7	0.993

Order Clostridiales	E2	0.06	0.49	26	0.993
Acetobacter	E2	0.03	0.518	11	0.993
Delftia	E2	0.02	0.588	7	1.000
Family Corynebacteriaceae	E2	0.02	0.598	10	1.000
Prevotella 2	E2	0.01	0.613	7	1.000
Phylum Saccharibacteria	E2	0.04	0.642	19	1.000
Dermabacter	E2	0.01	0.665	4	1.000
Dialister	E2	0.08	0.732	46	1.000
Parvimonas	E2	0.03	0.734	15	1.000
Pseudarcicella	E2	0.01	0.743	2	1.000
Alloprevotella	E2	0.00	0.749	2	1.000
Phylum Proteobacteria	E2	0.01	0.754	2	1.000
Ureaplasma	E2	0.01	0.763	2	1.000
Mobiluncus	E2	0.00	0.763	2	1.000
Propionimicrobium	E2	0.01	0.781	2	1.000
Acidaminococcus	E2	0.02	0.814	8	1.000
Peptococcus	E2	0.01	0.881	4	1.000
Campylobacter	E2	0.07	0.929	41	1.000
Veillonella	E2	0.45	0.96	323	1.000
Chryseobacterium	E2	0.01	0.961	6	1.000
Family Lachnospiraceae	E2	0.06	0.991	42	1.000
Neisseria	E2	0.00	1	4	1.000
Family Veillonellaceae	E2	0.00	1	3	1.000

Table S6. HMO concentrations and ratios among microbial lactotypes						
	Microbial lactotype					
HMO	L1	L2	L3	L4	*p*	FDR *p*
2'FL	4745±3294[a]	3315±2742[b]	3031±3360[bc]	2134±2444[c]	1.33E-07	1.13E-06
3FL	212±200	153±127	174±194	200±146	6.43E-02	7.54E-02
LNnT	825±489[a]	1006±543[b]	843±618[a]	870±366[ab]	7.82E-03	1.33E-02
3'SL	541±293	472±251	514±239	427±220	2.50E-02	3.40E-02
DFLac	338±266[a]	338±258[a]	318±303[a]	160±170[b]	1.33E-03	2.51E-03
6'SL	497±357[a]	451±214[a]	614±458[a]	368±397[b]	3.10E-04	8.11E-04
LNT	1508±1041	1648±980	1629±1229	1371±835	2.85E-01	2.93E-01
LNFP I	1132±1009	1140±1197	1138±1262	879±1071	1.22E-01	1.33E-01
LNFP II	1644±1101[ab]	1810±1228[a]	1328±993[b]	1729±1057[ab]	3.97E-02	5.00E-02
LNFP III	67±94[a]	31±41[b]	47±37[a]	44±55[ab]	1.08E-03	2.17E-03
LSTb	92±68	93±60	99±83	90±44	8.65E-01	8.65E-01
LSTc	142±121[a]	132±74[a]	174±135[a]	100±118[b]	4.08E-04	9.92E-04
DFLNT	1249±819[a]	939±766[b]	969±891[b]	701±759[b]	3.71E-05	1.58E-04
LNH	98±76	84±63	88±72	69±52	4.67E-02	5.67E-02
DSLNT	420±301[ab]	540±370[c]	441±276[ac]	317±189[b]	1.04E-03	2.17E-03
FLNH	48±52[a]	29±29[a]	46±66[a]	4±3[b]	4.84E-11	1.64E-09
DFLNH	134±129	106±109	96±86	88±99	2.33E-02	3.40E-02
FDSLNH	220±208[ab]	239±185[a]	215±221[ab]	166±200[b]	3.03E-02	3.97E-02
DSLNH	99±94[a]	108±78[a]	108±91[a]	49±46[b]	2.43E-04	7.51E-04
Total HMOs	14011±3736[a]	12634±3439[b]	11871±4558[b]	9768±3863[c]	3.71E-08	4.21E-07
Small HMOs	5995±3409[a]	4391±2767[b]	4333±3481[b]	3130±2349[b]	3.21E-07	2.18E-06
Modified lactose	238310±31136[a]	241837±37969[a]	225019±27337[b]	236339±27814[ab]	5.35E-03	9.58E-03
Type 1 HMOs	4796±2036	5230±2038	4635±2112	4385±1679	8.00E-02	9.06E-02
Type 2 HMOs	1034±548	1169±578	1063±660	1015±440	1.25E-01	1.33E-01
α1-2-fucosylated HMOs	5877±4005[a]	4455±3611[b]	4169±4049[bc]	3013±3426[c]	1.05E-05	5.08E-05
terminal α-2-6-sialylated HMOs	638±445[a]	582±265[a]	788±552[a]	469±479[b]	1.69E-04	5.74E-04
internal α-2-6-sialylated HMOs	511±342[a]	633±415[b]	540±328[ab]	406±213[a]	8.97E-03	1.45E-02
terminal α-2-3-sialylated HMOs	961±423[a]	1012±466[a]	955±400[a]	744±341[b]	1.48E-02	2.29E-02
HMO-bound Sia	2749±1188[a]	2921±1131[a]	2928±1238[a]	2049±1021[b]	2.87E-04	8.11E-04
HMO-bound Fuc	11511±4221[a]	9485±3568[b]	8745±5240[bc]	7056±3826[c]	8.67E-09	1.47E-07
HMO-bound Sia/total HMO	0.21±0.1[a]	0.24±0.09[b]	0.27±0.13[b]	0.22±0.09[ab]	4.57E-04	1.04E-03
HMO-bound Fuc/total HMO	0.81±0.17[a]	0.74±0.17[b]	0.69±0.25[b]	0.7±0.19[b]	2.55E-06	1.45E-05
HMO-bound Fuc/HMO-bound Sia	4.94±2.62[a]	3.72±1.86[b]	3.55±2.52[b]	4.19±2.92[ab]	6.31E-05	2.38E-04

Footnote: Concentrations are given as nmol/mL; mean ± sd. *P* values were calculated from Kruskal-Wallis test (followed by Dunn's post hoc test; letters denote significance groups, $p < 0.05$). 2'-fucosyllactose (2'FL), 3-fucosyllactose (3FL), lacto-N-neotetraose (LNnT), 3'-sialyllactose (3'SL), difucosyllactose (DFlac), 6'-sialyllactose (6'SL), lacto-N-tetraose (LNT), lacto-N-fucopentaose (LNFP) I, LNFP II, LNFP III, sialyl-LNT (LST) b, LSTc, difucosyllacto-LNT (DFLNT), lacto-N-hexaose (LNH), disialyllacto-N-tetraose (DSLNT), fucosyllacto-N-hexaose (FLNH), difucosyllacto-N-hexaose (DFLNH), fucodisialyllacto-lacto-N-hexaose (FDSLNH) and disialyllacto-N-hexaose (DSLNH); small HMOs (2'FL, 3FL, 3'SL, 6'SL, and DFLac), modified lactose (small HMOs and lactose), type 1 HMOs (LNT, LNFP I, LNFP II, LSTb, and DSLNT), type 2 HMOs (LNnT, LNFP III, and LSTc), α-1-2-fucosylated HMOs (2'FL

and LNFP I), terminal α-2-6-sialylated HMOs (6’SL and LSTc), internal α-2-6-sialylated HMOs (DSLNT and LSTb), terminal α-2-3-sialylated HMOs (3’SL and DSLNT), ratio of HMO-bound sialic acid (Sia) to total HMOs (HMO-bound Sia/total HMOs), ratio of HMO-bound fucose (Fuc) to total HMOs (HMO-bound Fuc/total HMOs), and ratio of the ratio of HMO-bound Fuc to HMO-bound Sia (HMO-bound Fuc/HMO-bound Sia).

Table S7. HMO concentrations and ratios between microbial enterotypes

	Enterotype			
HMO	E1	E2	*p*	FDR *p*
2'FL	3921±3314	3789±3125	6.96E-01	7.88E-01
3FL	213±200	160±144	8.74E-03	4.78E-02
LNnT	821±501	950±535	6.29E-03	4.28E-02
3'SL	539±286	468±241	9.84E-03	4.78E-02
DFLac	310±278	328±253	4.08E-01	6.60E-01
6'SL	502±386	480±316	5.10E-01	6.86E-01
LNT	1515±1078	1594±992	2.02E-01	4.02E-01
LNFP I	994±979	1271±1242	7.64E-02	2.16E-01
LNFP II	1655±1120	1618±1118	6.11E-01	7.42E-01
LNFP III	63±88	39±45	7.58E-02	2.16E-01
LSTb	92±72	95±59	2.31E-01	4.13E-01
LSTc	133±125	151±99	2.94E-03	3.34E-02
DFLNT	1128±808	1002±860	5.13E-02	1.94E-01
LNH	87±64	94±80	8.88E-01	8.93E-01
DSLNT	398±288	502±333	5.30E-04	1.80E-02
FLNH	42±55	34±40	4.64E-01	6.86E-01
DFLNH	124±123	106±106	2.48E-01	4.22E-01
FDSLNH	219±212	218±195	8.93E-01	8.93E-01
DSLNH	96±94	100±78	1.74E-01	3.92E-01
Total HMOs	12852±4192	12997±3829	7.68E-01	8.16E-01
Small HMOs	5174±3441	4897±3173	5.01E-01	6.86E-01
Modified lactose	236468±33410	237231±31015	5.24E-01	6.86E-01
Type 1 HMOs	4654±1959	5079±2095	6.74E-02	2.16E-01
Type 2 HMOs	1018±560	1139±569	1.23E-02	5.23E-02
α1-2-fucosylated HMOs	4914±3997	5060±3958	6.84E-01	7.88E-01
terminal α-2-6-sialylated HMOs	635±479	630±383	1.85E-01	3.92E-01
internal α-2-6-sialylated HMOs	490±330	597±375	1.91E-03	3.25E-02
terminal α-2-3-sialylated HMOs	937±424	970±432	7.18E-01	7.88E-01
HMO-bound Sia	2692±1237	2833±1118	8.69E-02	2.18E-01
HMO-bound Fuc	10231±4725	10001±4120	5.52E-01	6.95E-01
HMO-bound Sia/total HMO	0.23±0.11	0.23±0.09	4.55E-01	6.86E-01
HMO-bound Fuc/total HMO	0.77±0.2	0.75±0.18	8.99E-02	2.18E-01
HMO-bound Fuc/HMO-bound Sia	4.59±2.86	4.01±1.97	2.13E-01	4.02E-01

Footnote: Concentrations are given as nmol/mL; mean ± sd. 2'-fucosyllactose (2'FL), 3-fucosyllactose (3FL), lacto-N-neotetraose (LNnT), 3'-sialyllactose (3'SL), difucosyllactose (DFlac), 6'-sialyllactose (6'SL), lacto-N-tetraose (LNT), lacto-N-fucopentaose (LNFP) I, LNFP II, LNFP III, sialyl-LNT (LST) b, LSTc, difucosyllacto-LNT (DFLNT), lacto-N-hexaose (LNH), disialyllacto-N-tetraose (DSLNT), fucosyllacto-N-hexaose (FLNH), difucosyllacto-N-hexaose (DFLNH), fucodisialyllacto-lacto-N-hexaose (FDSLNH) and disialyllacto-N-hexaose (DSLNH); small HMOs (2'FL, 3FL,

3’SL, 6’SL, and DFLac), modified lactose (small HMOs and lactose), type 1 HMOs (LNT, LNFP I, LNFP II, LSTb, and DSLNT), type 2 HMOs (LNnT, LNFP III, and LSTc), α-1-2-fucosylated HMOs (2’FL and LNFP I), terminal α-2-6-sialylated HMOs (6’SL and LSTc), internal α-2-6-sialylated HMOs (DSLNT and LSTb), terminal α-2-3-sialylated HMOs (3’SL and DSLNT), ratio of HMO-bound sialic acid (Sia) to total HMOs (HMO-bound Sia/total HMOs), ratio of HMO-bound fucose (Fuc) to total HMOs (HMO-bound Fuc/total HMOs), and ratio of the ratio of HMO-bound Fuc to HMO-bound Sia (HMO-bound Fuc/HMO-bound Sia).
