# Supplementary figures and images for: Variation in Human Milk Composition Is Related to Differences in Milk and Infant Fecal Microbial Communities
Source: Microorganisms. 2021 May 27;9(6):1153. doi: 10.3390/microorganisms9061153 (PMC8230061; doi:10.3390/microorganisms9061153)

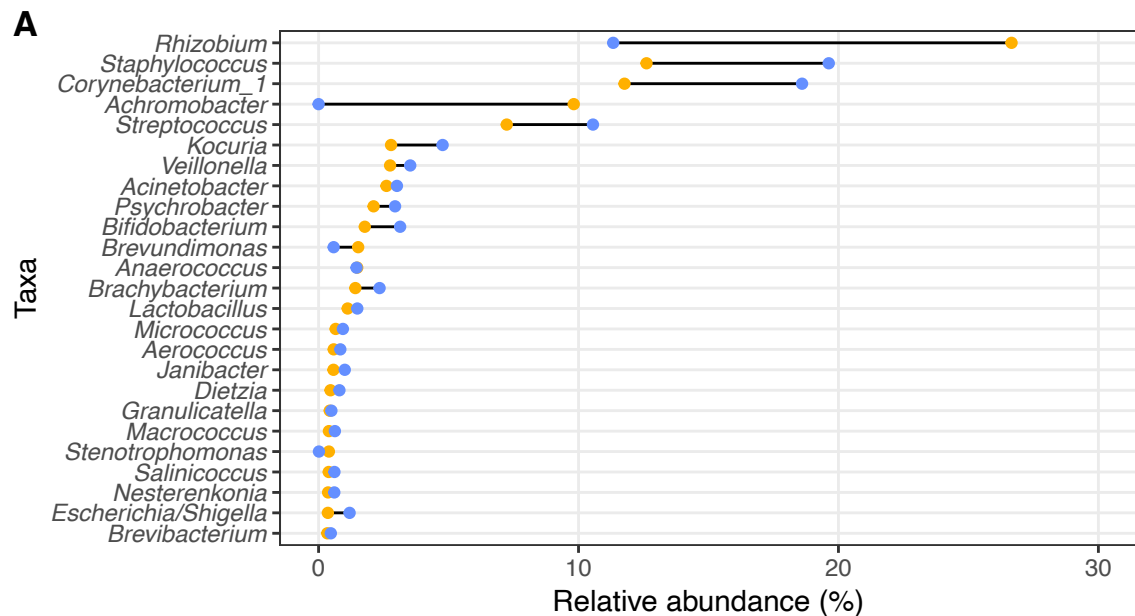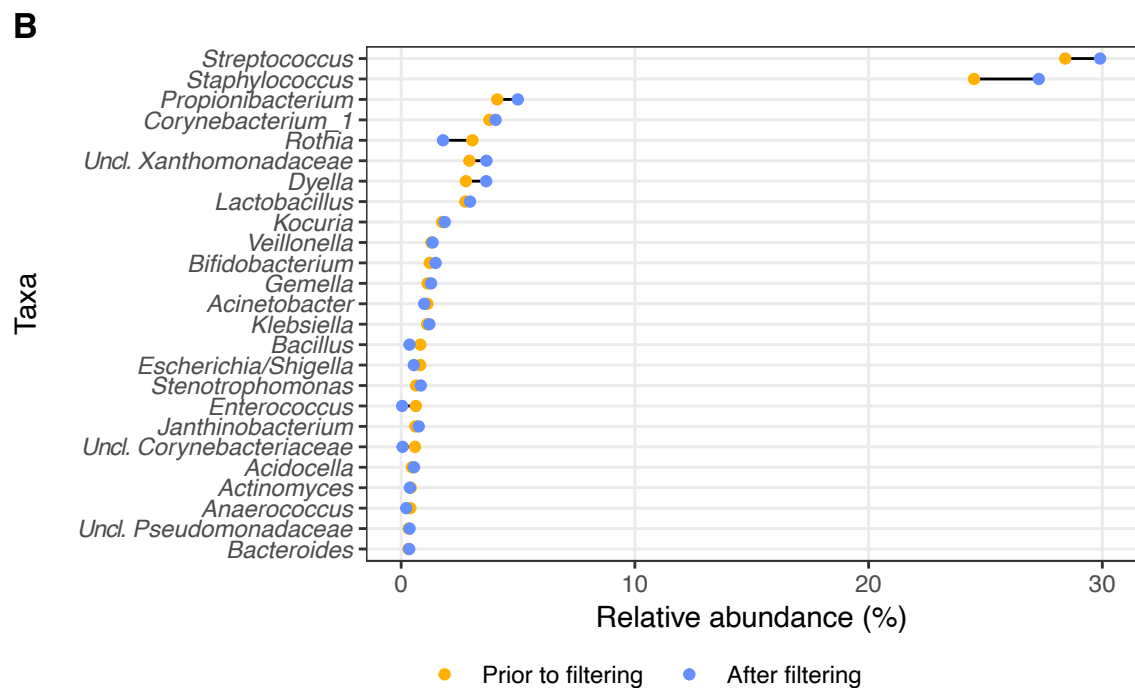

Supplement: Supplementary file 1 [file microorganisms-09-01153-s001.zip › FIGURE S1.pdf]

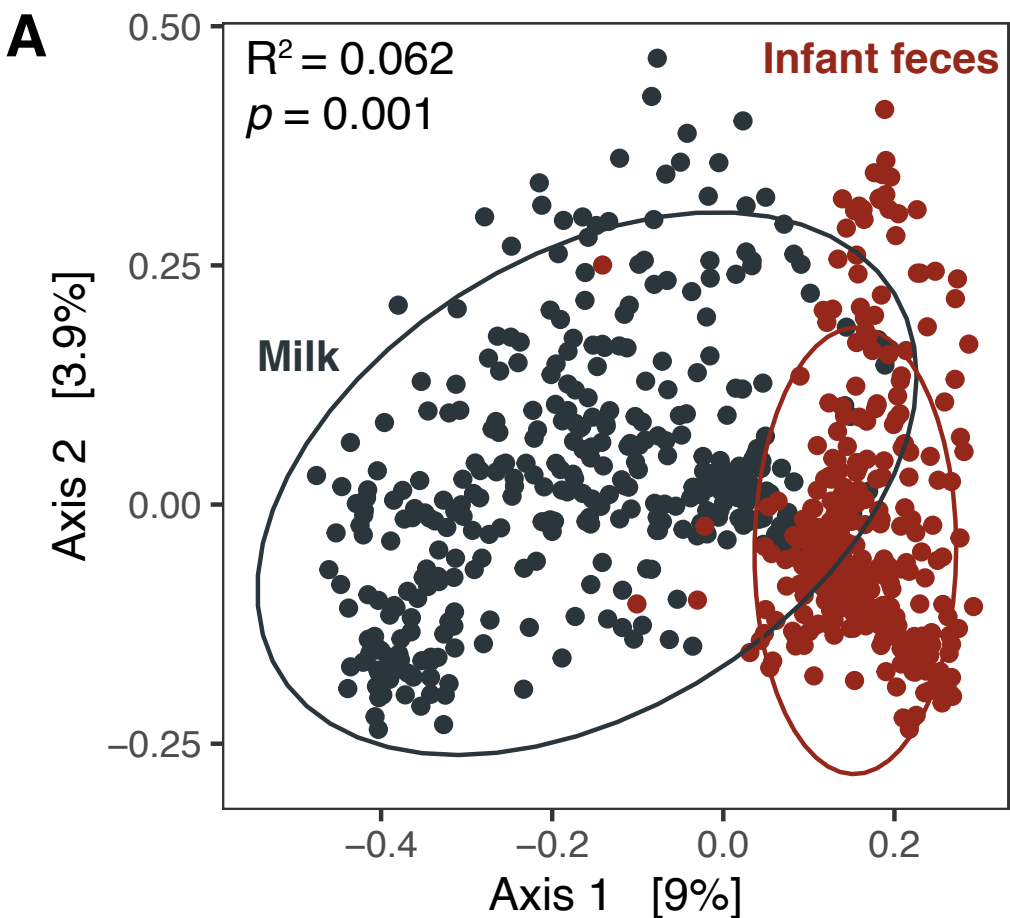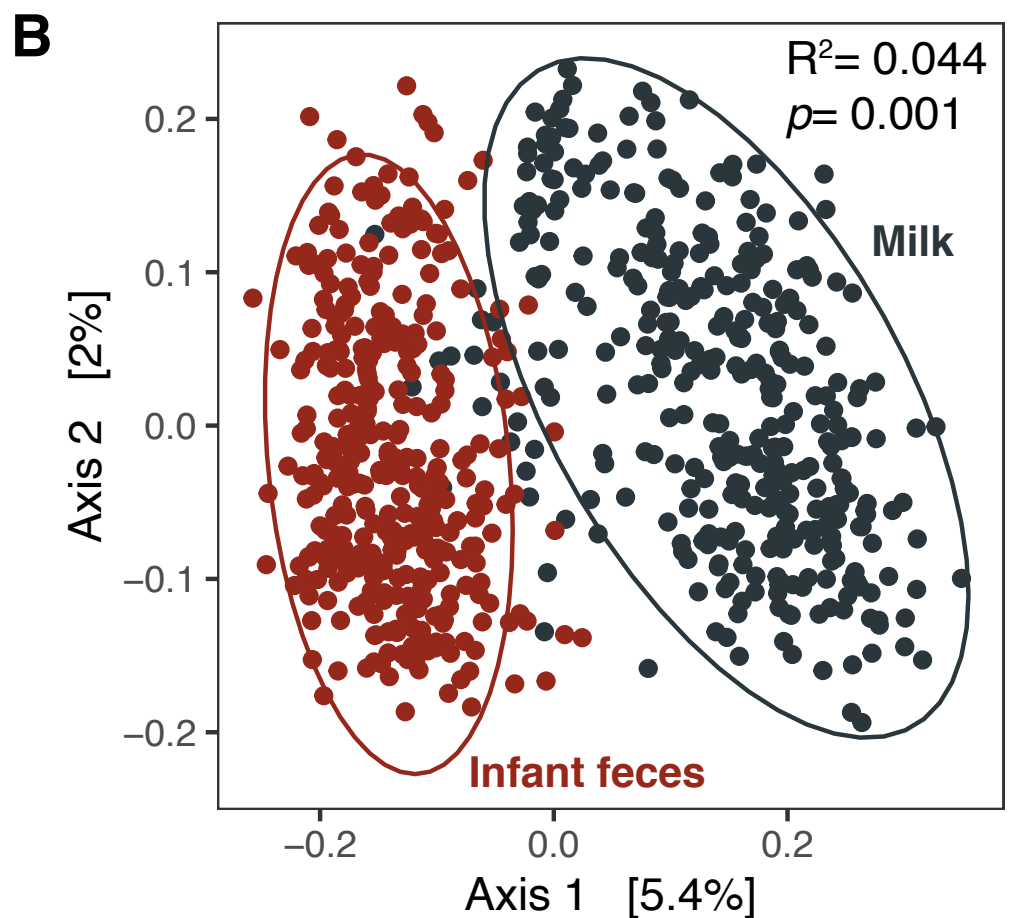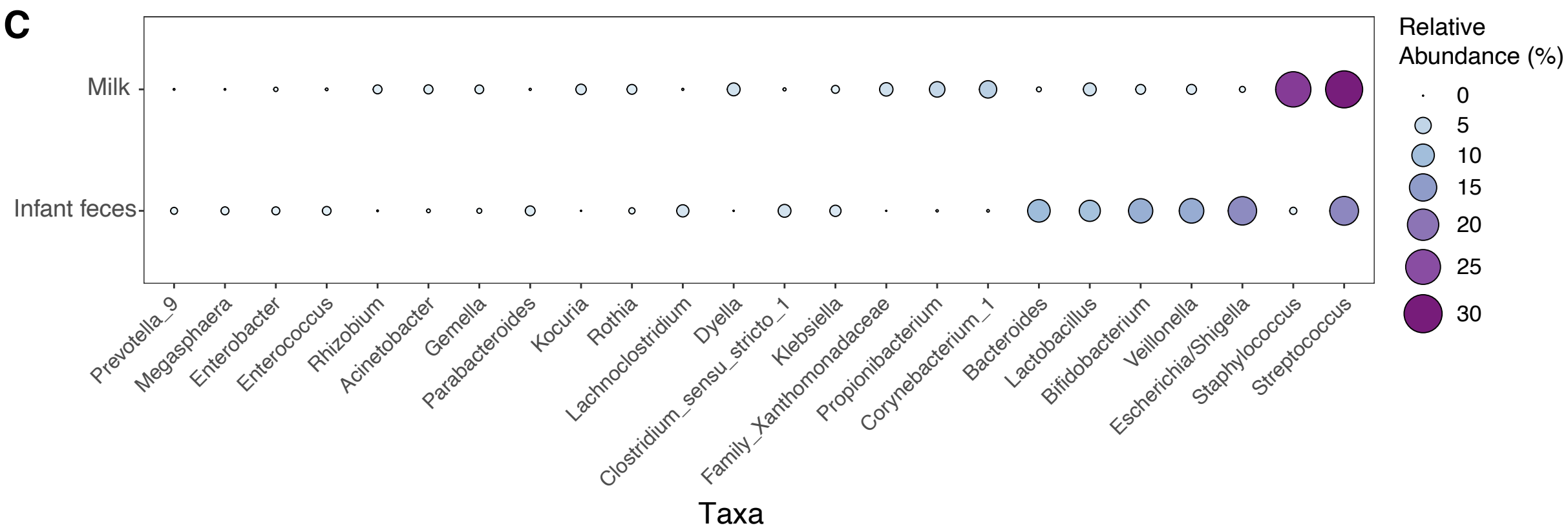

Supplement: Supplementary file 1 [file microorganisms-09-01153-s001.zip › FIGURE S2.pdf]

**A**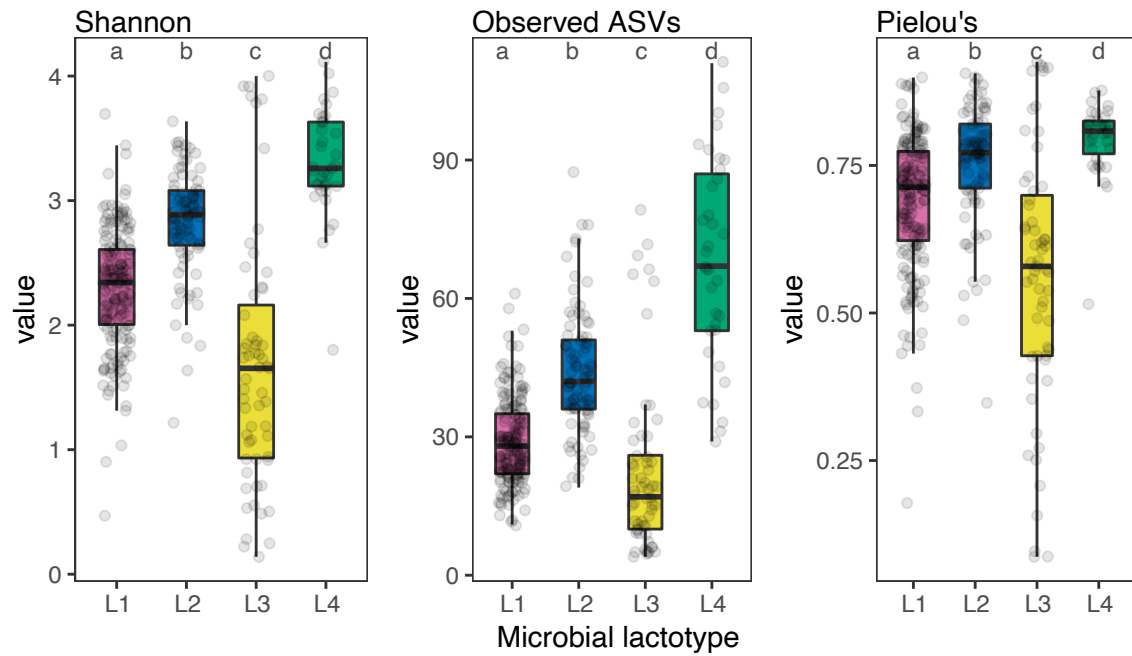**B**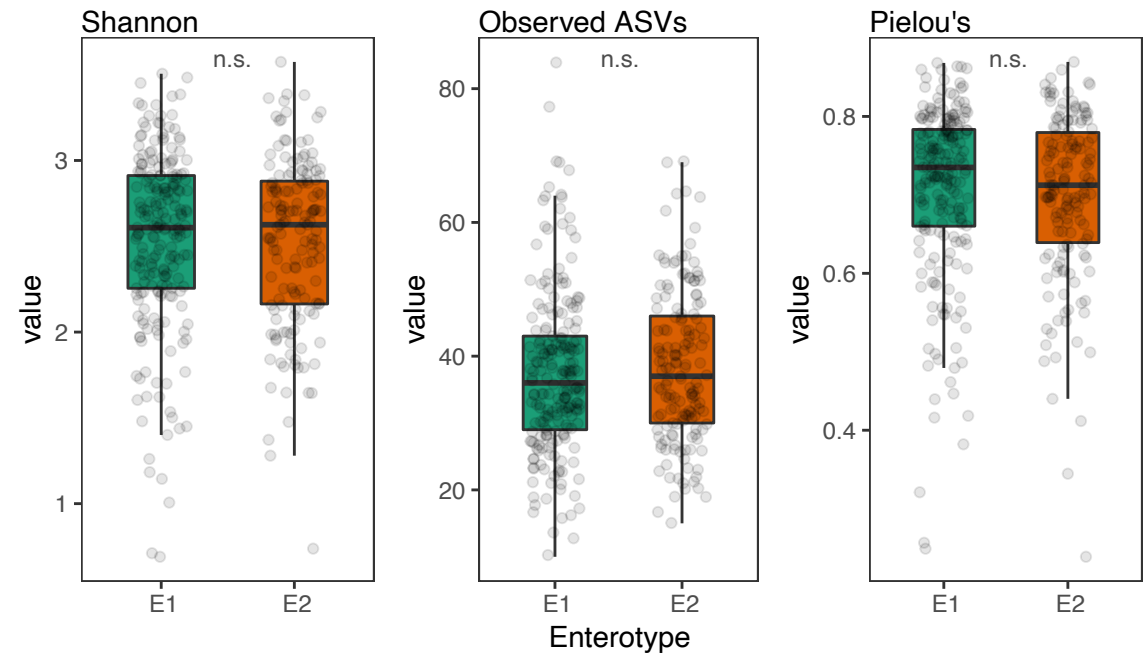**C**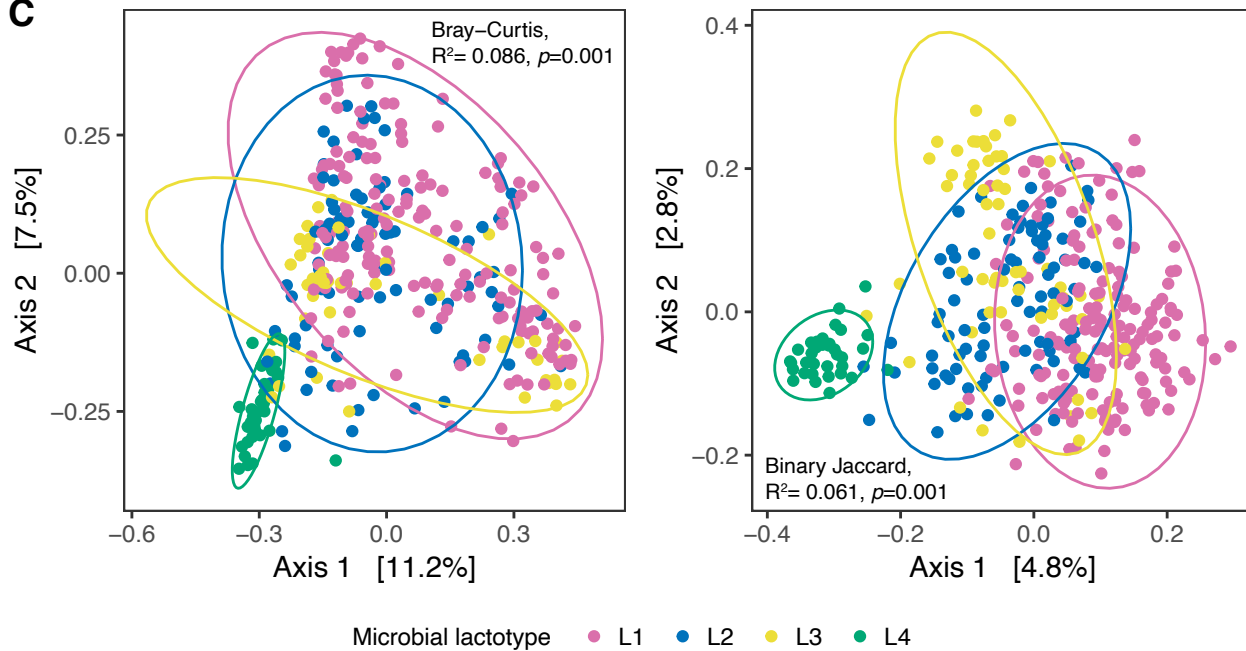**D**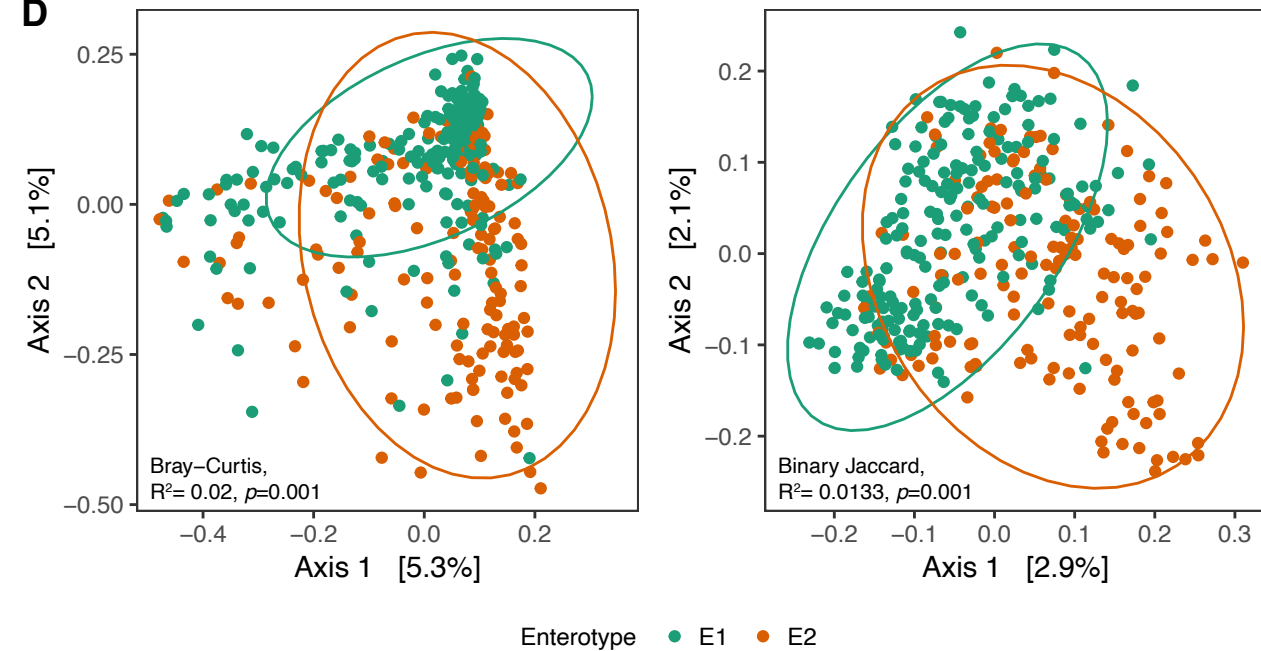

Supplement: Supplementary file 1 [file microorganisms-09-01153-s001.zip › FIGURE S3.pdf]

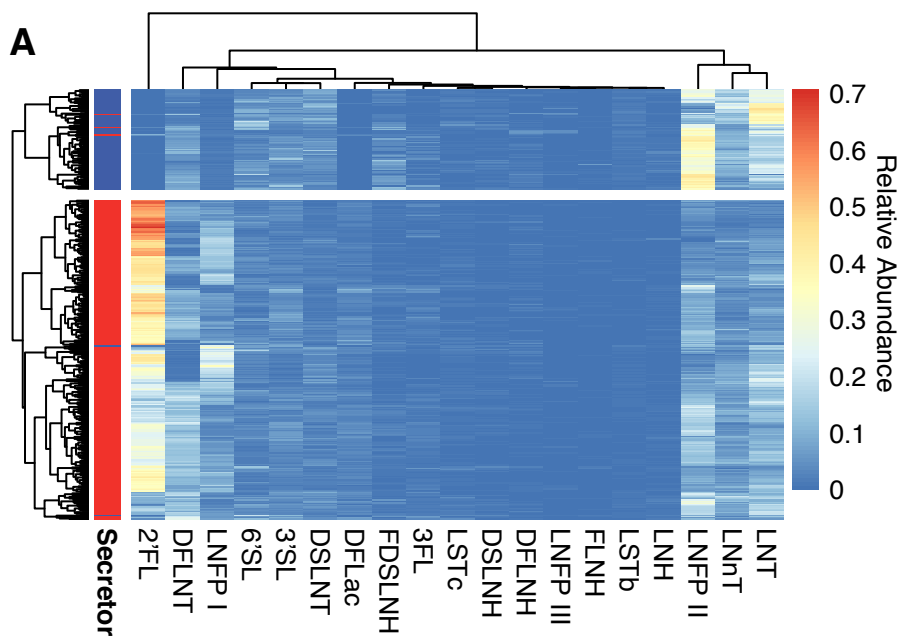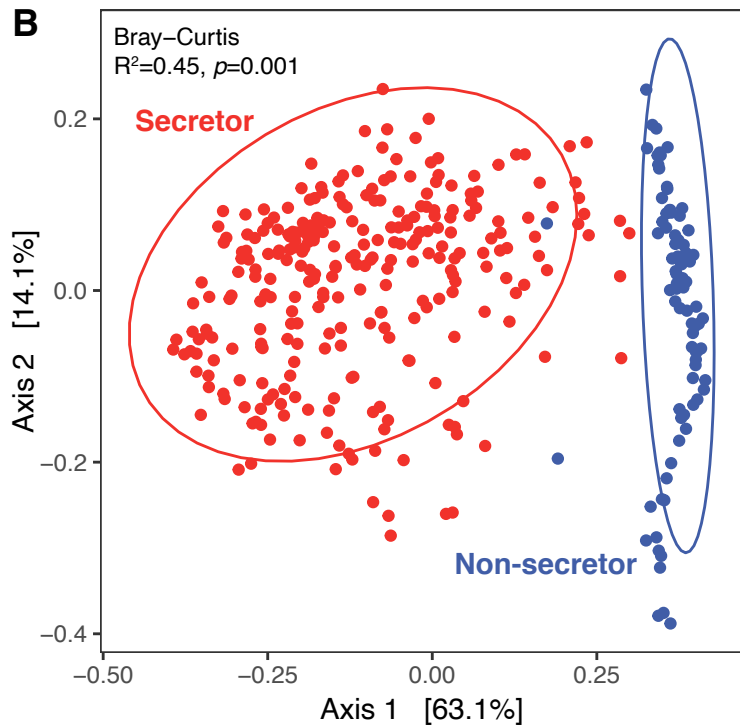

Supplement: Supplementary file 1 [file microorganisms-09-01153-s001.zip › FIGURE S4.pdf]
